# External Human–Machine Interfaces for Autonomous Vehicles from Pedestrians’ Perspective: A Survey Study

**DOI:** 10.3390/s22093339

**Published:** 2022-04-27

**Authors:** Jiawen Guo, Quan Yuan, Jingrui Yu, Xizheng Chen, Wenlin Yu, Qian Cheng, Wuhong Wang, Wenhui Luo, Xiaobei Jiang

**Affiliations:** 1 School of Mechanical Engineering, Beijing Institute of Technology, Beijing 100811, China; vera987654321@yeah.net (J.G.); yuanquan_0122@163.com (Q.Y.); yjryjr0522@163.com (J.Y.); xizhengchen@yeah.net (X.C.); 3120190450@bit.edu.cn (W.Y.); wangwh@bit.edu.cn (W.W.); 2 Chair of Ergomonics, Technische Universität München, Arcisstraße 21, 80333 Munich, Germany; billqiancheng@163.com; 3 Research Institute of Highway Ministry of Transport, Beijing 100088, China; wh.luo@rion.cn

**Keywords:** external human–machine interfaces, autonomous vehicle–pedestrian interaction, pedestrians’ safety, interface design

## Abstract

With the increasing number of automated vehicles (AVs) being tested and operating on roads, external Human–Machine Interfaces (eHMIs) are proposed to facilitate interactions between AVs and other road users. Considering the need to protect vulnerable road users, this paper addresses the issue by providing research evidence on various designs of eHMIs. Ninety participants took part in this experiment. Six sets of eHMI prototypes—Text, Arrowed (Dynamic), Text and Symbol, Symbol only, Tick and Cross and Traffic Lights, including two sub-designs (Cross and Do Not Cross)—were designed. The results showed that 65.1% of participants agreed that external communication would have a positive effect on pedestrians’ crossing decisions. Among all the prototypes, Text, and Text and Symbol, eHMIs were the most widely accepted. In particular, for elderly people and those unfamiliar with traffic rules, Text, and Text and Symbol, eHMIs would lead to faster comprehension. The results confirmed that 68.5% of participants would feel safer crossing if the eHMI had the following features: ‘Green’, ‘Text’, ‘Symbol’, or ‘Dynamic’. These features are suggested in the design of future systems. This research concluded that eHMIs have a positive effect on V2X communication and that textual eHMIs were clear to pedestrians.

## 1. Introduction

**Figure 1:**
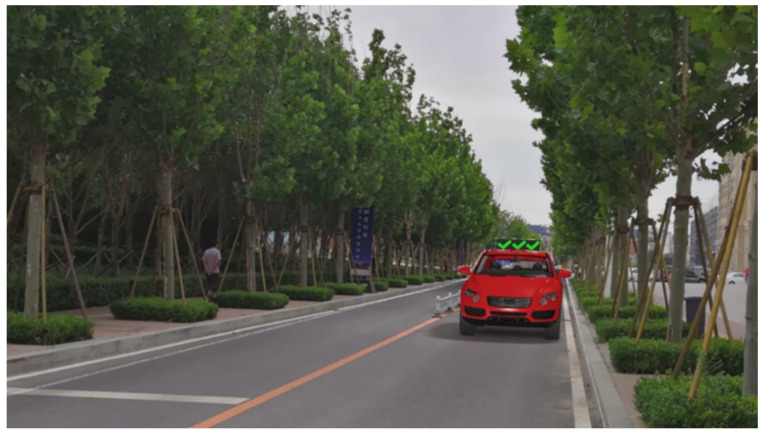
The cropped screenshots of the videos, showing the eHMIs used in this study for the roof position.

According to statistics, over 1.2 million people die in traffic accidents every year, and driver errors are a significant factor involved in this figure [1]. With the increasing number of automated vehicles (AVs) on roads, an emerging challenge concerns the interaction between AVs and non-automated road users such as pedestrians. AVs can be useful, and they do not require a driver to operate them, reducing the human workload and, thus, the number of car accidents [2]. Therefore, AVs have the potential to improve road safety [3].

In the road traffic environment, pedestrians feel safer by communicating with drivers [4,5,6,7]. However, with automated vehicles (AVs) of SAE Levels 4 (high driving automation) and 5 (full driving automation) [8], drivers are no longer required to control the vehicle at any time, and primary driving tasks are transferred to AVs. Drivers do not need to constantly monitor road conditions and interact with other road users through eye contact, gestures, lights, and horns while driving [9,10]. Vulnerable road users can determine the time that they have to cross the road by implicit communication, such as vehicle speed, vehicle distance, and other dynamic characteristics [11,12]; however, this can also remain ambiguous when a vulnerable road user encounters an approaching vehicle, especially if there is no zebra crossing or light signal. Therefore, it is necessary to develop new information feedback methods to compensate for the insufficient communication between vulnerable road users and drivers, especially in the initial stages of mixed traffic environments where pedestrians’ sense of comfort may be compromised due to concerns about technical safety [13]. Behavioral interaction between AVs and pedestrians will eventually replace driver–pedestrian communication; thus, this research is especially useful.

External Human–Machine Interfaces (eHMIs) convey the intention of the vehicle more clearly, which improves the efficiency of the interaction between AVs and road users. Explicit communication is recommended to compensate for the lack of implicit communication [5,14,15], especially in ambiguous or unregulated bottleneck scenarios, such as parking [16,17,18].

Previous studies have reported that, as a secondary source of information for pedestrians, eHMIs could affect pedestrians’ crossing decisions. Eye contact communication between pedestrians and AVs was established to test the effect of eHMIs [19]. The results showed that this eHMI significantly helped pedestrians feel safer, and pedestrians’ decision-making time decreased from 2.32 s to 2.03 s. In another study [20], an interface was designed to display the intentions of AVs to pedestrians. The pedestrians’ level of perceived safety and comfort was higher with the interface than without it. Based on experiences from a series of studies by Habibovic et al. [15], the overall conclusion was that such interfaces might be beneficial in situations where negotiation is needed. From a user survey with 29 participants, an eHMI design concept was proposed, and the results showed significantly shorter passing times and fewer crashes in the group with eHMIs [17].

However, the influence of implicit communication is still dominant [21]. In one experiment [22], all pedestrians crossed the road without explicit signaling, which suggested that pedestrians might not need explicit eHMI in routine interactions. Video-based observation and coding [23] also found that explicit communication was rare to nonexistent and that the motion patterns and behaviors of vehicles were more important for pedestrians when navigating efficient traffic negotiations. Pedestrians relied on legacy behaviors rather than leveraging the information on an external display [24].

Different methods have been introduced in studies focusing on the effect of eHMIs, e.g., images, videos, Wizard of Oz (WoZ), virtual reality (VR), and the Delphi method. Images were used to compare 30 eHMI concepts within a short space of time [25]. Similarly, 729 images were generated with an eHMI to explore appropriate colors for eHMIs. Compared to images, videos can show the dynamic status of vehicles more directly, which is beneficial for obtaining real reactions [23,26]. The WoZ approach has a relatively high degree of authenticity and can reproduce the pedestrian crossing scenario to the greatest extent. In WoZ studies, the human driver hides behind a seat cover and drives the vehicle along predefined trajectories repeatedly [27,28,29]. However, study time, weather, traffic conditions, and strict ethical requirements limit the application of WoZ. According to immersion and interactivity, VR technology has been used in many studies [20,30]. However, VR headsets may cause discomfort or even dizziness and influence the crossing behavior of pedestrians. The Delphi method was used in eHMI analyses. Sixteen human factor researchers were interviewed [21] about their personal perspectives on automated vehicles (AVs) and their interaction with VRUs in the future environment.

The problem ‘what kind of information should be displayed on eHMIs and how’ was considered. Four categories of information were suggested for the design [14]: (1) information about vehicle driving status, (2) information about AV’s intention, (3) information about AV’s perceptions of the environment, and (4) information about AV’s cooperation capabilities. In addition, some comparison studies were conducted. The effect of no eHMI, status eHMI, status + perception eHMI, status + intent eHMI, and status + perception + intent eHMI was investigated [31]. The status + intent eHMI was considered the ideal interface to enhance user experience [29,31,32]. Moreover, pedestrians found that egocentric eHMI messages were clearer than allocentric messages and adopted an egocentric perspective if the message was ambiguous [33]. In addition, an 18-dimensional classification was proposed to construct the description of the eHMI concept [34], which mainly covers the key attributes of the eHMI from two different aspects: physical characteristics and its usability and realism.

The features of the eHMIs’ visual displays, including the category, characteristics, and location of the information, were studied. Results from one study [25] showed that text displays stating “Walk” or “Don’t Walk” were clear to pedestrians, whereas green or red headlights were relatively ambiguous. Other studies also found that eHMls increased the efficiency of pedestrian–AV interactions, and textual eHMIs were considered the clearest form of communication [35,36]. Regarding nontextual eHMIs, a zebra crossing symbol was effective, whereas light-based eHMIs were relatively fuzzy. When designing the eHMIs’ color [37], green is suggested when the AV is yielding. Green and red should not be used if the AV is not yielding. Various colors could be used for that purpose, including cyan, yellow, and purple.

In summary, the literature proposes a large variety of designs for eHMIs. eHMIs were evaluated in terms of text, color, anthropomorphism, and perspective, through different methods. However, to the best of our knowledge, studies on the preferences of different populations for eHMIs should be taken into account. Exploring this motivated our research effort. In this study, 6 sets of eHMIs with 12 prototypes, applied to a road segment with mid-block crosswalk, were designed, and they were interpreted during user interviews. By collecting the demographic characteristics of the participants and their evaluation of eHMI concepts, the design elements of eHMIs and users’ preferences regarding the messages’ presentation format were analyzed.

## 2. Materials and Methods

### 2.1. Methodology

User interviews were conducted to analyze the design elements of an eHMI based on the understanding and experience of road users. At the beginning of the interview, the demographic characteristics of the participants were provided, and anonymity and voluntary participation were explained to the participants.

Questionnaires with multiple-choice lists, Likert scales, and open-ended question series were used for the interviews. Demographic information was collected, including gender, age, with/without driver’s license, and basic knowledge of traffic rules and AVs. Common sense questions about traffic rules and AVs, such as “Choose the best explanation for the given traffic signs”, “How much do you think you know about autonomous driving”, and others, were implemented to measure the basic knowledge range of the respondents. Basic knowledge of traffic rules and AVs were classified into four (‘extremely familiar’, ‘familiar’, ‘unfamiliar’, and ‘extremely unfamiliar’) and five groups (‘extremely familiar’, ‘fairly familiar’, ‘familiar’, ‘not really familiar’, and ‘extremely unfamiliar’), according to the answers of questions about traffic rules and AVs.

The major questionnaire focused on the design of eHMIs. Six sets of eHMI prototypes were designed based on existing prototypes in the literature and redesigned according to Chinese culture. Each set consisted of two sub-designs: the participants have the right-of-way (in green), and the participants should yield (in red) (Table 1). Cinema 4D R20 was used to make an animated simulation of the encountering scenario, and the frame rate of the animation was 25 Hz. The height of the camera was 1.7 m with a 45° angle to the zebra crossing to show the general visual angle of participants crossing the street. The vehicle decelerated from an initial speed of 30 km/h, with a deceleration of −1.6 m/s^2^ from 30 m, and stopped in front of the zebra crossing for approximately 5 s. To avoid a learning effect, respondents were asked to judge the intention of the vehicle from the eHMI information. Figure 1 shows a screenshot of a video of the scenario. For each video, participants gave a score to evaluate their general feeling and understanding of the eHMI. This included ‘The eHMI information *N* is visually clear and self-explanatory to me’; ‘The eHMI information *N* strengthens my confidence when crossing’; and a score of user experience, ranging on a 5-point Likert scale from (1) ‘extremely dissatisfied’ to (5) ‘extremely satisfied’. After the general score was collected, the perspectives on the eHMI prototypes, focusing on the acceptable design elements and preferred interaction modes, were collected by the open-ended questions.

### 2.2. Participants

Ninety participants took part in this survey; there were five invalid responses, so the final sample consisted of 44 males and 41 females. The participants were a minimum of 9 years of age (Mage = 37.24 years, SDage = 2.44 years), whose guardians’ consent for their participation were given. People in this age range are thought to possess the cognitive skills to facilitate successfully crossing the street [38], and these skills are important for road users. Additionally, none of them had constraints in terms of normal or corrected-to-normal vision.

### 2.3. Data Analysis

Descriptive statistics for gender, age, with/without driver’s license, and basic knowledge of traffic rules and AVs can be found in Table 2. Participants’ basic knowledge of traffic rules and AVs was divided into four and five grades, respectively, according to the accuracy of the correlation questions. In addition, pedestrians’ evaluations on the yielding behavior of AVs were also statistically described.

Each Likert scale rank to evaluate eHMI was assigned a number, ranging from ‘strongly dislike’ = 1 to ‘strongly prefer’ = 5. We reported the percentage of respondents in each category that strongly dislike or prefer the Likert scale items. Kendall’s tau-b correlation analysis was used because age and basic knowledge of traffic rules/AVs are ordered categorical variables and others are unordered categories variables. Additionally, the results (<0.3) revealed that there was no significant correlation among these factors affecting the eHMIs’ preference. Then, to test the differences among the types of eHMI and factors, nonparametric statistical tests, such as the Friedman test, were used because the data were not normally distributed and categorical variables.

## 3. Results

### 3.1. Overall Evaluation of eHMIs

**Figure 2:**
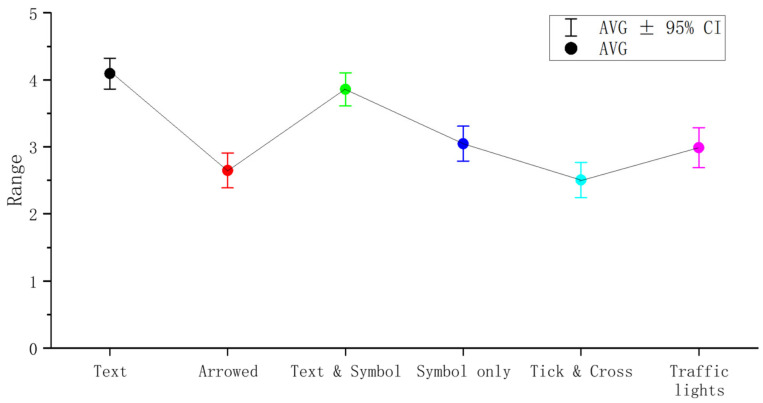
An overall evaluation ranking (‘strongly dislike’ = 1 to ‘strongly prefer’ = 5) of different eHMIs, presented through boxplots.

The overall evaluation of different eHMIs on a five-point scale (from low to high) was analyzed statistically, as shown in Figure 2. According to the evaluation scores, from high to low, the results were as follows: Textual eHMI (M = 4.09, SD = 1.065), Textual and Symbolic eHMI (M = 3.86, SD = 1.313), Symbolic eHMI (M = 3.05, SD = 1.204), the ‘Traffic Lights’ eHMI (M = 2.99, SD = 1.384), Arrowed eHMI (M = 2.65, SD = 1.212), and the ‘Tick and Cross’ eHMI (M = 2.51, SD = 1.211).

The distribution of ranks conformed with a normal distribution (*p* < 0.05). Then, a Friedman test was chosen to compare the differences between the ranks of different eHMIs. The tested hypothesis was that the probability distribution of eHMIs was the same. The results revealed that there was a significant difference among different types of eHMI (*χ*^2^ = 118.733, *p* = 0.000 < 0.05). To explore the specific divergences between them, Bonferroni-adjusted *p* values were used for pairwise comparison of the Friedman test, as shown in Table 3 below.

Accordingly, the evaluation scores of Textual eHMI and Textual and Symbolic eHMI were significant, but there was no divergence among the other four categories (Arrowed eHMI, Symbolic eHMI, the ‘Tick and Cross’ eHMI, and the ‘Traffic Lights’ eHMI). Although the Textual eHMI type interface was relatively more user-friendly to the majority of participants, the preferences for the eHMI among different pedestrian groups were undetermined.

By using the Kruskal-Wallis H test, the effects of social and demographic characteristics on different eHMI prototypes were investigated, as shown in Table 4.

**Figure 3:**
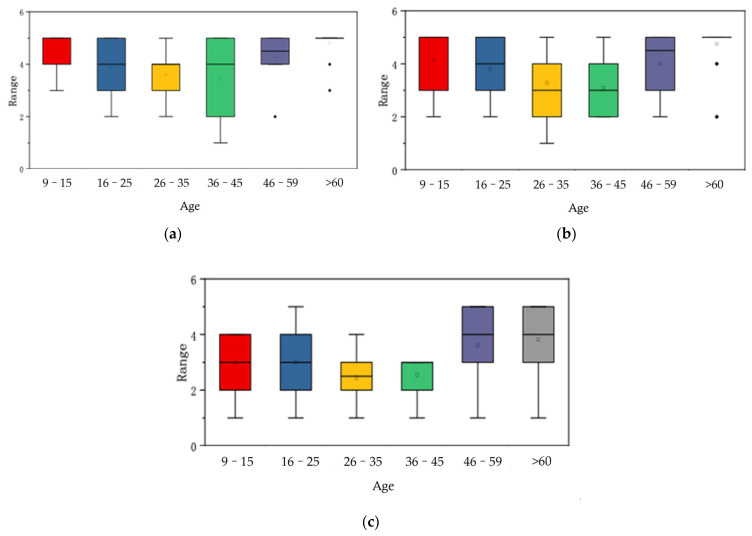
The rank distribution of eHMIs: (**a**) Textual eHMI, (**b**) Textual and Symbolic eHMI, and (**c**) Symbolic eHMI in different age groups, presented through boxplots.

**Figure 4:**
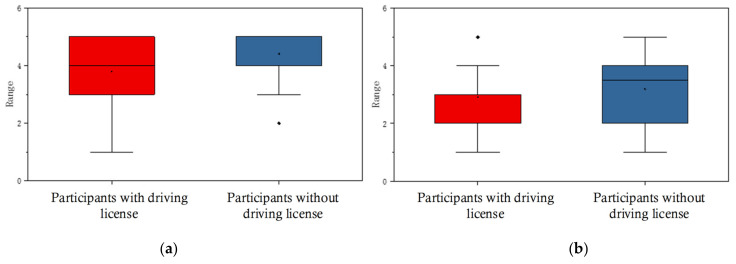
The rank distribution of eHMIs: (**a**) Textual eHMI; (**b**) Textual and Symbolic eHMI in With/Without driver’s license groups, presented through boxplots.

**Figure 5:**
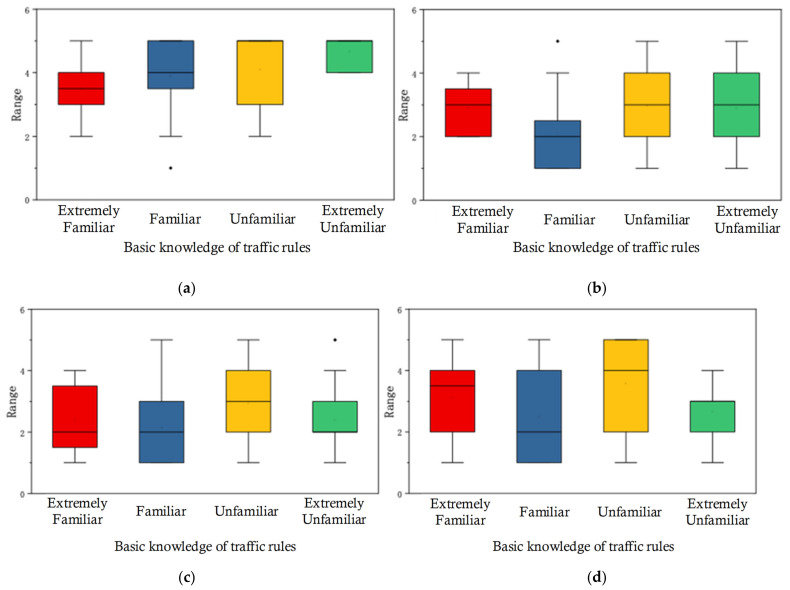
The rank distribution of eHMIs: (**a**) Textual eHMI, (**b**) Arrowed eHMI, (**c**) Tick and Cross eHMI, and (**d**) Traffic Lights eHMI in terms of basic knowledge of traffic rules, presented through boxplots.

There were no significant differences among all eHMIs regarding gender (male and female) and basic knowledge of AVs (all *p* > 0.05). However, for age, differences in the evaluation ranks of eHMIs (Textual eHMI, Textual and Symbolic eHMI, and the ‘Traffic Lights’ eHMI) were significantly higher (*p* < 0.05). Regarding driver’s license, considerable differences were found in the Textual eHMI and Symbolic eHMI groups. Moreover, basic knowledge of traffic rules affected pedestrians’ evaluation of the Textual eHMI, Textual and Symbolic eHMI, the Arrowed eHMI, and the ‘Tick and Cross’ eHMI (*p* < 0.05). As with age, with/without driver’s license and basic knowledge of traffic rules had an influence on the evaluation; eHMI porotypes were compared, and the data were visualized, as shown in Figure 3, Figure 4 and Figure 5.

In terms of age, the Kruskal-Wallis test of independent samples was carried out for the three aforementioned groups. The test revealed that people over the age of 60 preferred the Textual eHMI compared to younger participants (16–25, 26–35, and 36–45; *p* < 0.05). The older participants (>60) rated Textual and Symbolic eHMI highly compared with the 26–35 and 36–45 groups (*p* < 0.05). For Symbolic eHMIs, there were significant differences between the evaluations of the older (>60) and 26–35 group (*p* < 0.05). To conclude, eHMIs such as Textual eHMI, Textual and Symbolic eHMI, and Symbolic eHMI were highly valued by the older participants (>60).

Significant differences (*p* < 0.05) were found in the evaluation scores of the Textual eHMI and Textual and Symbolic eHMI. Figure 4 illustrates the relationship between driving license and the evaluation scores. It reveals that people without a driver’s license clearly preferred eHMI with Textual eHMIs and with Textual and Symbolic eHMIs.

To establish participants’ familiarity with traffic rules, a Kruskal-Wallis test of independent samples was carried out for four groups. Figure 5 shows the ranks from the groups with different levels of traffic rules familiarity. For the Textual eHMI, pedestrians who were extremely unfamiliar with traffic rules tended to give a high appraisal compared with those who were slightly unfamiliar (*p* < 0.05). Regarding the Arrowed eHMI, ‘Tick and Cross’ eHMI, and ‘Traffic Lights’ eHMI, the evaluation given by the unfamiliar group was significantly more acceptable than the evaluation given by the familiar group (*p* < 0.05).

### 3.2. Considerations of Pedestrians When Making Crossing Decisions

**Figure 6:**
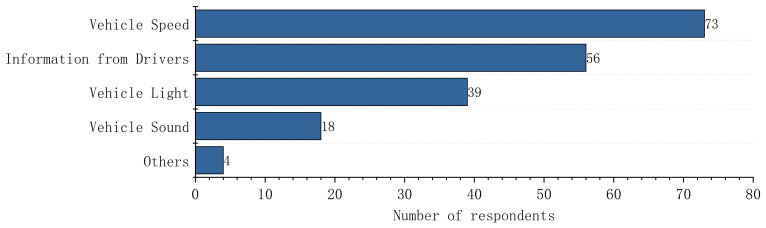
The considerations of pedestrians when making crossing decisions.

Figure 6 shows the considerations of pedestrians when making crossing decisions. AV speed was chosen to have considerable importance by 85.89% (*N* = 73) of the participants. A total of 65.9% (*N*_Female_ = 30, *N*_Male_ = 26) preferred to receive information via explicit communication, such as drivers’ gestures and language. Vehicle light (45.9%, *N*_Female_ = 17, *N*_Male_ = 22) and sound (21.2%, *N*_Female_ = 10, *N*_Male_ = 8) were also important to pedestrians.

**Figure 7:**
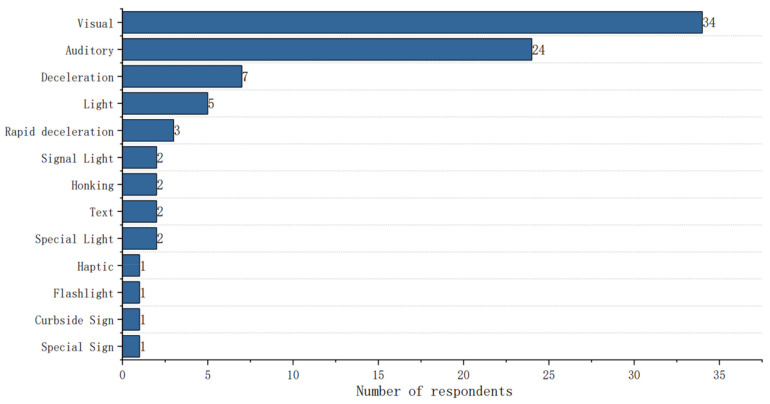
The word frequency statistics of preferred interaction modes between AVs and pedestrians.

**Figure 8:**
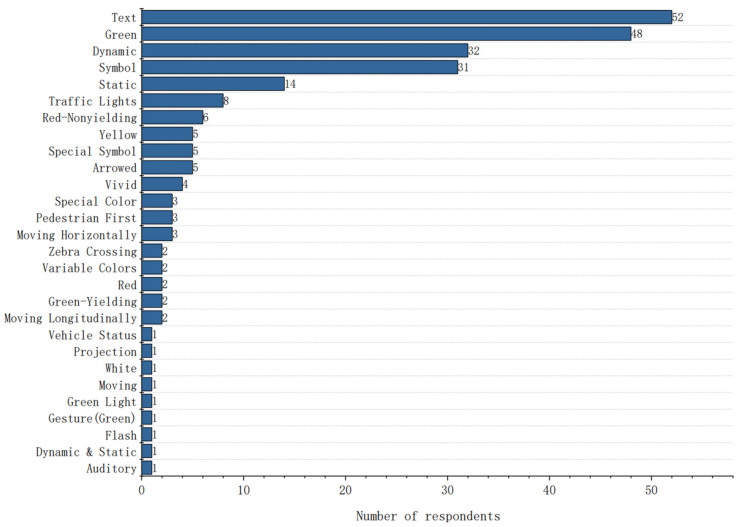
The word frequency statistics of acceptable design elements of eHMIs.

In the open-ended question series, the design elements of the eHMI and the AV–pedestrian interaction modes were discussed according to their preferences, and participants chose the interaction modes between AVs and pedestrians. The results are depicted as word frequency statistics in Figure 7 and Figure 8. 

Figure 7 illustrates that participants had a strong preference for the use of visual interaction (*N* = 34) integrated with auditory stimuli when encountering AVs. Auditory eHMIs were recommended by 24 participants, and auditory interactions could raise pedestrians’ awareness and elicit a shorter reaction time [39,40,41], but it was uncertain whether auditory eHMIs caused distractions. Additionally, pedestrians might be confused when encountering vehicle platoons with auditory eHMIs. Light (*N* = 5) was considered to be an interaction mode only if it was standardized. As haptic eHMIs, portable devices such as mobile phones were suggested to interact with pedestrians.

Figure 8 indicates that explicit communication through the color green (*N* = 48) and text eHMIs (*N* = 52) was highly acceptable. Additionally, symbols (*N* = 31) were regarded as an intuitive characteristic compared to text because of their higher visual definition. The dynamic interface (*N* = 32) was more perceivable than the static interface (*N* = 14) as pedestrians could see it clearly from a greater distance. Furthermore, traffic lights (*N* = 8) were advised to be used in the eHMIs.

## 4. Discussion

### 4.1. Information via eHMI Design

This study aimed to investigate the differences between six sets of eHMI prototypes, which benefited a standardized narrative in designing future eHMIs. The Textual eHMI and Textual and Symbolic eHMI were the most acceptable designs. The Textual eHMI, Textual and Symbolic eHMI, and Symbolic eHMI transferred information to elderly pedestrians more clearly than to younger pedestrians. Furthermore, the Textual eHMI and Textual and Symbolic eHMI were more user-friendly for pedestrians who were unfamiliar with traffic rules. Textual eHMIs, providing yielding or non-yielding information directly, appeared to require no explicit learning. The learning cost of the Symbolic eHMIs, which were derived from traffic lights for pedestrians, was also small. Smaller learning costs lead to a shorter cognitive processing time for pedestrians’ understanding, as pointed out by another study [35]. However, the Arrowed, Tick and Cross, and Traffic Lights eHMIs were considered to provide no explicit instruction. Participants needed more time to determine whether the eHMIs were designed from an egocentric perspective or from an allocentric visual perspective. Accidents may occur if participants’ judgments are not consistent with the designer’s purpose.

Approximately 65.9% of pedestrians believed eHMIs would be a part of the interaction system for AVs and vulnerable road users. The speed information of AVs can be added to future eHMIs as 85.89% of pedestrians’ crossing decisions were affected by the AV’s speed. The results indicated that it was possible to display the speed of the AV on future eHMIs. Audio was a highly accepted interaction mode. Light and haptic eHMIs were also regarded as effectual modes. For a well-designed eHMI, those with green text color such as ‘Pedestrian First’ were suggested to show the intention of yielding. Symbols such as traffic lights were ambiguous, which might mislead pedestrians into making dangerous decisions. However, specifically designed symbols with simple instructions or short-term training were recommended for the yielding scenarios.

### 4.2. Limitations and Future Studies

Limitations regarding the size of the sample, diversity of the participants, and sensitivity analysis will be resolved in future research. According to some existing studies [18,24], participants’ experience with AVs and participants’ nationality [42] may also influence their decision when interacting with AVs. Cultural differences and an understanding of specific colors/symbols/expressions among different regions will be considered in future work. The installation position of the eHMI will also be measured because of its effects on pedestrians’ crossing decisions [41]. Additionally, the physiological condition of pedestrians, like heartbeats synchronized with dynamic events, will be considered to impact non-conscious errors in the control of dynamic events [43]. In addition, only the eHMI adapted to the full self- driving vehicles (L5-level) was studied in this research. For L3/L4-level AVs, further studies should be conducted considering human–systems dissonances about decision conflicts between drivers and systems [2].

## 5. Conclusions

This research invited ninety participants to take part in a survey comparing six sets of eHMIs. Differences in pedestrians’ preferences for eHMIs were found. The effects of age and basic knowledge of traffic rules were large for the text and symbol eHMIs. Textual and Text and Symbol eHMIs, which significantly increase the efficiency of pedestrians’ crossing decisions, are widely accepted, especially for elderly people (>60) or people who are unfamiliar with traffic rules. Different response biases were observed among users with basic knowledge of traffic rules. We found that respondents unfamiliar with traffic rules are more likely to give positive responses to the Arrowed eHMI, the ‘Tick and Cross’ eHMI, and the ‘Traffic Lights’ eHMI. Furthermore, certain types of eHMIs displaying the yielding situation with the characteristics of ‘Green’, ‘Text’, ‘Symbol’, or ‘Dynamic’ were proposed by the vast majority; these are suggested in the design of future human–AV interaction systems.

This research studied the preferences of different populations in the design of eHMIs, considering the influences of demographic factors, basic knowledge of traffic rules and AVs, etc. It contributes design considerations of eHMIs for people with different characteristics, and it is also helpful in terms of improving the acceptance of autonomous vehicles especially for different people. In addition, this research will benefit the design of eHMIs and provide suggestions regarding improvements.

## Figures and Tables

**Table 1:** eHMIs for AVs proposed by researcher.

Design Elements	Sets of eHMI Prototypes	
Text *	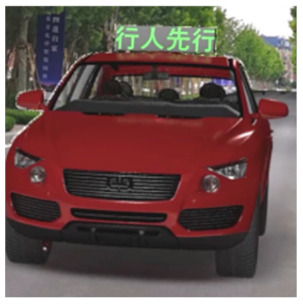	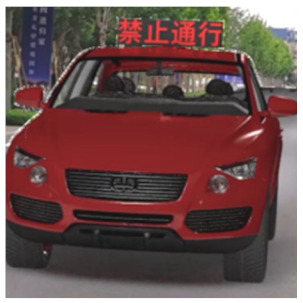
Arrowed	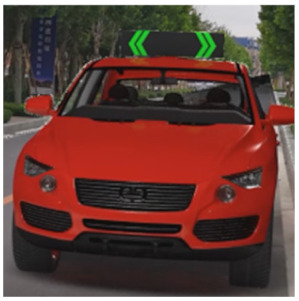	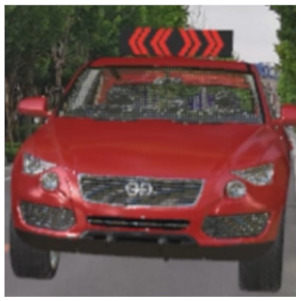
Text and Symbol	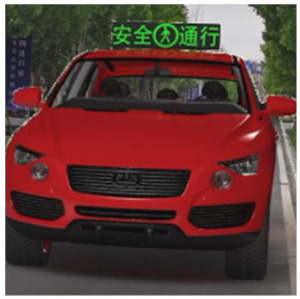	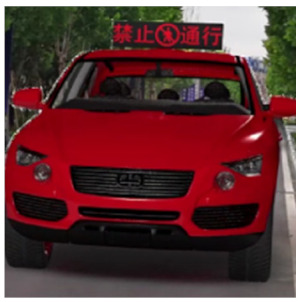
Symbol Only	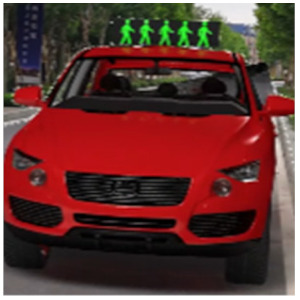	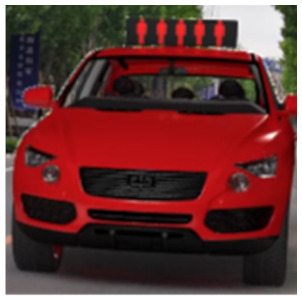
Tick and Cross	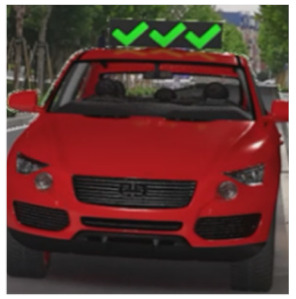	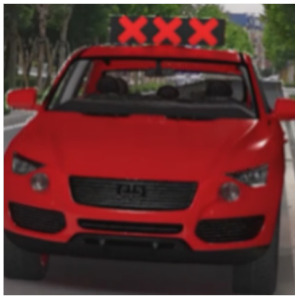
Traffic Lights	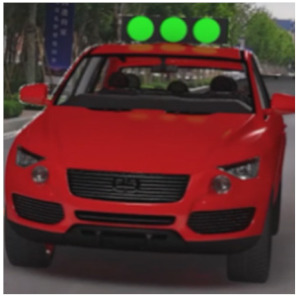	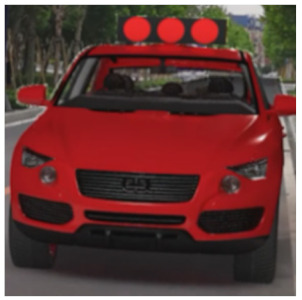

* The Chinese characters was used to design the textual eHMI prototypes.

**Table 2:** A summary of demographic data, including gender, age, with/without driver’s license, and basic knowledge of traffic rules and AVs.

Characteristics	Value	Summary Statistics: %
Gender	Male	51.8%
	Female	48.2%
Age	9–15 years old	15.3%
	16–25 years old	20.0%
	26–35 years old	21.2%
	36–45 years old	12.9%
	46–59 years old	11.8%
	>60 years old	18.8%
With/Without driver’s license	Yes	50.6%
	No	49.4%
Basic knowledge of traffic rules	Extremely Familiar	9.4%
	Familiar	32.9%
	Unfamiliar	36.5%
	Extremely Unfamiliar	21.2%
Basic knowledge of AVs	Extremely Familiar	1.2%
	Fairly Familiar	9.4%
	Familiar	12.9%
	Not Really Familiar	58.9%
	Extremely Unfamiliar	17.6%

**Table 3:** The *p* values of six types of pairwise comparative analyses. If *p* < 0.05, the evaluation scores between factors were significant.

Design Element	Text	Arrowed	Text and Symbol	Symbol only	Tick and Cross	Traffic Lights
Text	-	<0.05	>0.05	<0.05	<0.05	<0.05
Arrowed	<0.05	-	<0.05	>0.05	>0.05	>0.05
Text and Symbol	>0.05	<0.05	-	<0.05	<0.05	<0.05
Symbol only	<0.05	>0.05	<0.05	-	>0.05	>0.05
Tick and Cross	<0.05	>0.05	<0.05	>0.05	-	>0.05
Traffic Lights	<0.05	>0.05	<0.05	>0.05	>0.05	-

**Table 4:** The *p* values of six types under different characteristics. If *p* < 0.05, the evaluation scores between factors were significant.

Design Element	Text	Arrowed	Text and Symbol	Symbol Only	Tick and Cross	Traffic Lights
Gender	>0.05	>0.05	>0.05	>0.05	>0.05	>0.05
Age	<0.05	>0.05	<0.05	<0.05	>0.05	>0.05
With/Without driver’s license	<0.05	>0.05	<0.05	>0.05	>0.05	>0.05
Basic knowledge of traffic rules	<0.05	<0.05	>0.05	>0.05	<0.05	<0.05
Basic knowledge of AVs	>0.05	>0.05	>0.05	>0.05	>0.05	>0.05

## Data Availability

Not applicable.
